# Neuropsychological functions as trait markers in OCD: a long term follow-up

**DOI:** 10.1192/j.eurpsy.2022.497

**Published:** 2022-09-01

**Authors:** M. Puialto, J. Segalàs

**Affiliations:** Bellvitge Hospital, Psychiatry, Barcelona, Spain

**Keywords:** OCD, Neuropsychology, ObssesiveCompulsiveDisorder, Long term follow-up

## Abstract

**Introduction:**

There is suggestive evidence that Obssesive Compulsive Disorder (OCD) is characterized by impaired neuropsychological functions that are also influenced by clinical variables. Several studies show that these neuropsychological deficits could be potential endophenotype markers.

**Objectives:**

The present study aimed to examine neuropsychological patterns in OCD patients and several clinical variables before and after a follow-up of 10 years.

**Methods:**

This study examined 44 outpatients with OCD. Cognitive performance and clinical data of these patients were documented before and after a follow-up of 10 years. A neuropsychological battery was administered and scored to them including Rey Osterrieth Complex Figure, the Digit-span test, and the State-Trait Anxiety Inventory. As well, several clinical variables were also assessed including sociodemographic variables, general intelligence measured by Progressive Raven’s matrices, Yale Brown Obsessive Compulsive Scale and Hamilton Depression Rating Scale. Finally, data was analyzed using t-Student and Pearson’s correlation.

**Results:**

In general, the pattern of neuropsychological dysfunction in patients with OCD remains unchanged during the follow-up period, except for some specific variables. Low scores on some verbal memory tasks were associated with severity of OCD, and nonverbal memory was influenced by depressive symptoms in the first evaluation, while, after the follow-up, as obsessive and affective symptoms improve, there’s no significant change in the neuropsychological pattern.

**Conclusions:**

Despite the influence of some clinical and sociodemographic variables on the neuropsychological performance in OCD patients, cognitive dysfunction remains unchanged after a follow-up period of 10 year. These results suggest that cognitive deficits could be considered as a trait marker for the disorder.

**Disclosure:**

No significant relationships.

